# Estimating Soil Organic Carbon Stocks and Spatial Patterns with Statistical and GIS-Based Methods

**DOI:** 10.1371/journal.pone.0097757

**Published:** 2014-05-19

**Authors:** Junjun Zhi, Changwei Jing, Shengpan Lin, Cao Zhang, Qiankun Liu, Stephen D. DeGloria, Jiaping Wu

**Affiliations:** 1 College of Environmental and Resource Sciences, Zhejiang University, Hangzhou, China; 2 Ocean College, Zhejiang University, Hangzhou, China; 3 Department of Crop and Soil Sciences, Cornell University, Ithaca, New York, United States of America; DOE Pacific Northwest National Laboratory, United States of America

## Abstract

Accurately quantifying soil organic carbon (SOC) is considered fundamental to studying soil quality, modeling the global carbon cycle, and assessing global climate change. This study evaluated the uncertainties caused by up-scaling of soil properties from the county scale to the provincial scale and from lower-level classification of Soil Species to Soil Group, using four methods: the mean, median, Soil Profile Statistics (SPS), and pedological professional knowledge based (PKB) methods. For the SPS method, SOC stock is calculated at the county scale by multiplying the mean SOC density value of each soil type in a county by its corresponding area. For the mean or median method, SOC density value of each soil type is calculated using provincial arithmetic mean or median. For the PKB method, SOC density value of each soil type is calculated at the county scale considering soil parent materials and spatial locations of all soil profiles. A newly constructed 1∶50,000 soil survey geographic database of Zhejiang Province, China, was used for evaluation. Results indicated that with soil classification levels up-scaling from Soil Species to Soil Group, the variation of estimated SOC stocks among different soil classification levels was obviously lower than that among different methods. The difference in the estimated SOC stocks among the four methods was lowest at the Soil Species level. The differences in SOC stocks among the mean, median, and PKB methods for different Soil Groups resulted from the differences in the procedure of aggregating soil profile properties to represent the attributes of one soil type. Compared with the other three estimation methods (i.e., the SPS, mean and median methods), the PKB method holds significant promise for characterizing spatial differences in SOC distribution because spatial locations of all soil profiles are considered during the aggregation procedure.

## Introduction

Soil organic carbon (SOC), which plays a critical role in the global carbon cycle, comprises a major part of the terrestrial carbon reservoir [1]–[3]. In terrestrial ecosystems, SOC stock is almost three times the size of carbon storage in the vegetation of terrestrial ecosystems [4], and approximately twice as large as carbon storage in the atmosphere [5]. Because of the important role of SOC and its large quantity stored in terrestrial ecosystems, a slight change in SOC stock may influence global climate [6]–[8]. Accurately quantifying SOC stock has become a focus of present research on global climate change, and is considered essential for studying soil quality, modeling the global carbon cycle, and assessing global climate change [8]–[12].

Different SOC stock estimates, however, can vary greatly at both global and regional scales [13]–[17]. For global scales, Bohn [18] estimated the total SOC stock was 3,000 Pg (1 Pg = 10^15 ^g), whereas Bolin [19] estimated only 710 Pg, over a four-fold difference. In China, estimated SOC stocks for terrestrial ecosystems range from 50 Pg [20] to 185.7 Pg [21], also approximately a four-fold difference. The methodology for estimating SOC stocks, such as soil profile-based [22]–[24] and model-based (e.g., the CENTURY [25] and DeNitrification-DeComposition models [26]), is one of the main factors attributed to the wide range of differences in SOC stock estimation from different studies. The soil profile-based methodology calculates SOC stock using soil profiles and their corresponding areas obtained from soil survey products (the soil type method), vegetation type maps (the vegetation type method), or life zone maps (the life zone method), among which the soil type method is the most widely used [9], [18]–[19], [27]–[28]. According to the sources of soil area, the soil type method contains the Soil Profile Statistics (SPS) method and the GIS-based Soil Type (GST) method [2]. The SPS method calculates SOC stock by multiplying the SOC density value of a soil type by its corresponding field survey area recorded in soil survey reports (e.g., Soil Species of China [29]). The GIS-based Soil Type method calculates areas of various soil types accurately based on digital soil map and can provide information on the spatial distribution of SOC stocks.

In China, different scale (i.e., county, provincial, and national) soil survey products (e.g., soil survey reports, soil maps) of the Second National Soil Survey of China are the most important data sources for SOC stock estimations [15], [20]–[21], [30]–[33]. For the Second National Soil Survey of China, soil profile properties were aggregated sequentially in the county, provincial, and national scales. Different number of sampling profiles can be used to represent the attributes of one soil type at different scales and can cause uncertainties in SOC stock estimation. At the provincial scale, calculating the mean and median of SOC density values of multiple soil profiles of the same soil type name to represent the SOC density value of that soil type are the two most commonly used GIS-based Soil Type methods [32]–[34]. At the county scale, the SPS method [20], [30]–[32] and the pedological professional knowledge based (PKB) method [2], [15], [34]–[36] are the two most commonly used methods. Compared with the median or mean method, the PKB method aggregates soil profile properties downscaling from the provincial scale to more detail soil map units, which links a SOC density value of each soil profile to a digital soil map according to the identity or similarity in soil parent materials and spatial locations of all soil profiles at the county scale. However, the uncertainties of soil profile properties caused by up-scaling from the county scale to the provincial scale among the four methods remain unknown.

Soil map up-scaling includes soil classification level up-scaling and resolution up-scaling. The former aggregates Soil Species to higher soil classification levels (e.g., Soil Group, Subgroup, Soil Family); the latter aggregates higher resolution soil maps to lower resolution ones, such as from 1∶50,000-county-scale map to 1∶1,000,000-country-scale map. Both the soil classification level up-scaling and resolution up-scaling can cause uncertainties in SOC stock estimation. Zhao et al. [34] tested the effects of soil map resolution up-scaling from 1∶500,000 to 1∶10,000,000 using the three GIS-based Soil Type methods (i.e., the mean, median, and PKB methods) on SOC stock estimation for Hebei Province, China. However, the effects of these three GIS-based Soil Type methods on SOC stock estimation at soil map resolution larger than 1∶500,000 remain unknown. Moreover, the uncertainties in SOC stock estimation caused by soil classification level up-scaling using the SPS, mean, median, and PKB methods remain unknown.

The goal of this study is to evaluate the uncertainties of changes in scale among the four estimation methods (i.e., the SPS, mean, median, and PKB methods) by using a newly completed 1∶50,000 soil survey geographic database of Zhejiang Province, China. Specifically, the main objectives of this study were to: (1) evaluate the uncertainties caused by up-scaling of soil profile properties from the county scale to the provincial scale and soil classification levels from Soil Species to Soil Group among the four methods; and (2) quantify spatial differences in SOC stock estimation among the three GIS-based Soil Type methods.

## Materials and Methods

### Study Area

**Figure 1:**
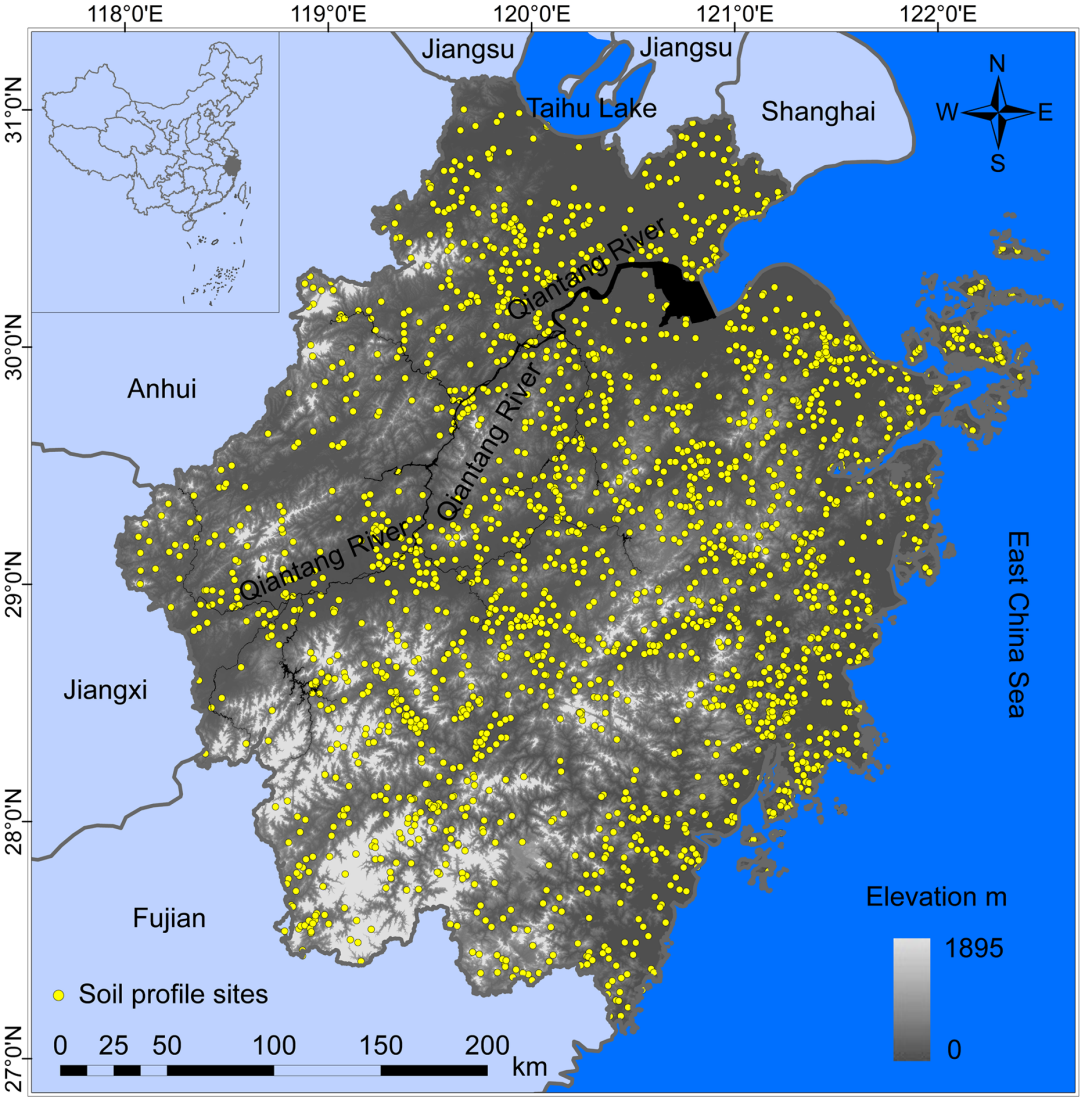
Study area and distribution of soil profile sites.

Zhejiang Province is located between 27°06′ and 31°03′ N latitude and 118°01′ and 123°10′ E longitude in southeastern China (Figure 1). The Province covers a land area of 1.02*10^5^ km^2^. Hills and mountains are dominant terrain, occupying 70.4% of the total land area. The plains and basins account for 23.2%, and the remaining 6.4% is comprised of lakes and rivers. Provincial topography is characterized by high mountains in the southwest and low plains in the northeast. The elevation ranges from 0 to 1895 m with an average of 296 m above sea level.

With a subtropical monsoon climate, Zhejiang Province has an annual average temperature from 15 to 18°C and an annual precipitation of 1200 to 1800 mm. As substantial differences exist in climate, geomorphology, geohydrology, land use, and parent material throughout the Province, its vegetation and soils and their spatial distribution patterns vary greatly. According to Soils of Zhejiang Province [37], Red soils are the dominant Soil Group, accounting for approximately 39% of the total area of Zhejiang soils.

### Data Sources

A recently completed 1∶50,000 soil survey geographic database of Zhejiang Province [38] was used in the study, which is the most detailed soil survey data at the provincial scale in China to date. The database includes Soil Spatial Data and Soil Attributes Data. The Soil Spatial Data include a 1∶50,000 digital soil map of the Province and a digital map of 2154 geo-referenced soil sampling profile sites, both of which were derived by digitizing and re-compiling field soil survey maps at 1∶50,000 from 76 counties in Zhejiang. The Soil Attributes Data contain the properties of the 2154 geo-referenced soil sampling profiles, which were taken from original county soil survey reports. For each profile, there are one to seven soil layers; for each layer, there are up to 104 soil physical and chemical properties, including geographic location, depth, bulk density, organic matter content, and gravel content, etc.

The soil survey geographic database was used to estimate SOC stocks with the mean, median, and PKB methods. This information from the Second National Soil Survey of China conducted in early 1980’s was the most comprehensive and detailed inventory of soil characteristics in Zhejiang Province. The Genetic Soil Classification System of Zhejiang Province was used during the field soil survey. The soils were classified using a six-level hierarchical scheme: Order, Suborder, Group, Subgroup, Family, and Species. The Soil Species is the basic classification level, and the Soil Group is the most commonly used classification level in China [2]. According to the Genetic Soil Classification System of Zhejiang Province, the collected 2154 soil profiles originated from 10 Soil Groups, 21 Subgroups, 99 Soil Families and 277 Soil Species.

### Estimation of Organic Carbon Density for Soil Profiles

For each of the 2 154 soil profiles, SOC density was calculated with the formula [34], [39]:
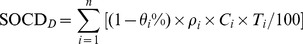
(1)where SOCD*_D_* (kg m^−2^) is the SOC density of a soil profile within a depth of *D* (cm), *n* is the number of soil layers in the soil survey, *θ_i_*% represents the volumetric percentage of gravel (>2 mm) content, *ρ_i_* is the soil bulk density (g/cm^3^), *C_i_* is the organic carbon content (C g/kg), and *T_i_* represents the thickness (cm) of the layer *i*. Density of SOC was estimated to a maximum depth of 100 cm to facilitate comparison among data sets. For profiles with actual depths greater than or equal to 100 cm, but less than 100 cm was observed, data for the unobserved profile sections were derived from the mean values of all the corresponding soil profiles of the same Soil Species or Soil Family [15], [33], [40]. Organic carbon content is calculated by multiplying soil organic matter content by 0.58 (the Bemmelen index), which is based on the assumption that soil organic matter contains approximately 58% organic carbon [41].

### Estimation of Regional Soil Organic Carbon Stocks

The SOC stocks of the Province were estimated by using the SPS, mean, median, and PKB methods (Table 1).

**Table 1:** Methods used to estimate SOC (soil organic carbon) stocks in Zhejiang Province, China.

Method	SOC densityvalue	Scale	Note
SPS^a^	Mean	County; soilspecies	(1) One soil species has multiple areas, which were surveyed countyby county. (2) Mean SOC density value was calculated from oneor multiple profiles within the county. (3) 2154 soil profiles were used.
Mean ormedian	Mean or median	Province; soilspecies	(1) One soil species has one area calculated from the digital soil map.(2) Mean or median SOC density value was calculated from oneor multiple profiles within the Province. (3) 2154 soil profiles were used.
PKB^a^	Mean	County; soilmap unit	(1) Soil map units were derived from the digital soil map countyby county. (2) One soil map unit in one county may have oneor multiple areas calculated from the digital soil map. (3)Mean SOC density value was calculated from one ormultiple profiles located within one polygon; polygonsbelong to one soil map unit in one county mayassigned different SOC density values. (4)2154 soil profiles were used.

a SPS, Soil Profile Statistics; PKB, pedological professional knowledge based.

For the SPS method, the mean SOC density value of each soil type in one county was calculated from one or multiple profiles belonging to that soil type; then the SOC stock value for that soil type was calculated by multiplying its SOC density value by its corresponding area; finally, the SOC stock values of all soil types were summed up as the total SOC stock for the Province. The area calculated from the 1∶50,000 digital soil map was used in this study to facilitate comparison among different methods.

For the mean or median method, the arithmetic mean or median of SOC density values of all soil profiles of the same soil type name in the Province was calculated first as the SOC density value of that soil type; secondly, SOC stocks for polygons on the 1∶50,000 digital soil map were calculated by multiplying the mean or median values by corresponding areas of the soil types and then were summed up as the total stock for the Province.

For the PKB method, the SOC density value of each of the 2154 soil profiles was linked to corresponding polygons on the 1∶50,000 digital soil map according to the same soil type name, the soil parent materials, and the spatial locations by county. When two or more soil profiles of the same soil type name were located in one polygon, the mean SOC density value of these soil profiles was calculated and used for the linkage. The SOC stock values for all polygons were calculated and then were summed up as the total stock for the Province.

The estimation of SOC stocks using the three GIS-based Soil Type methods were performed using ESRI software ArcGIS 10.0 (Redlands, CA) with water and urban areas excluded from the calculation.

## Results

### Effect of Soil Classification Levels Up-Scaling from Soil Species to Soil Group on SOC Estimation Using Different Methods

**Figure 2:**
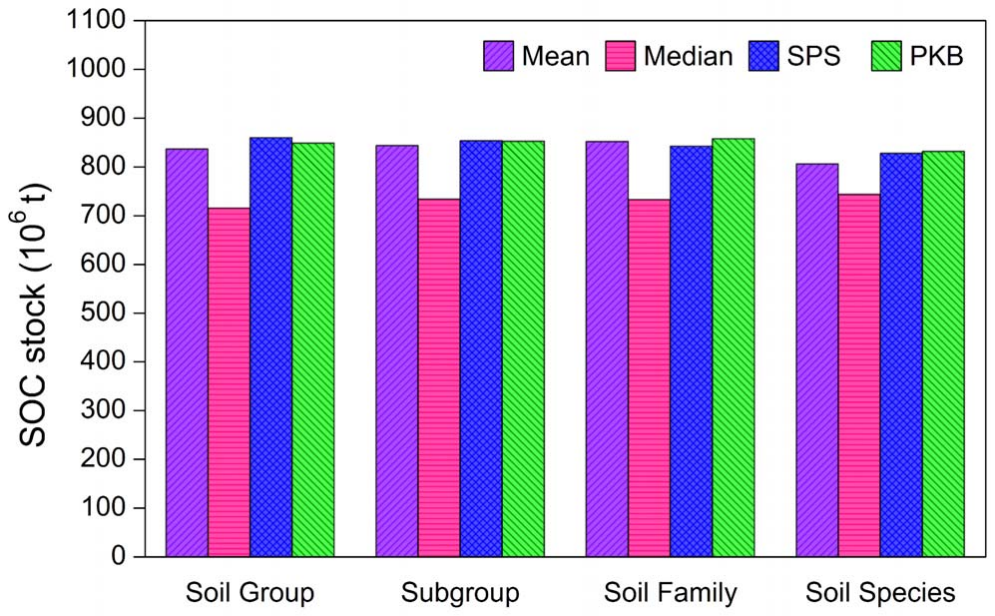
The estimates of SOC (soil organic carbon) stocks for soil classification levels up-scaling from Soil Species to Soil Group using the mean, median, SPS (soil profile statistic), and PKB (pedological professional knowledge based) methods.

The SOC stocks for soil classification levels up-scaling from Soil Species to Soil Group using the mean, median, SPS, and PKB methods were calculated (Figure 2). Among the four methods, the estimated SOC stocks for the study area using the median method were always the lowest regardless of soil classification level. Because the SOC densities of the 2154 soil profiles showed a positively skewed distribution with skewness of 14.17 (Table 2), the estimated SOC stocks using the mean method were much higher than those using the median method at all four soil classification levels.

**Table 2:** Descriptive statistics of profile soil organic carbon densities by Soil Group.

Soil Group	Soil Order of U.S. Taxonomy^a^	N^b^	Range	Min^b^	Max^b^	Mean	Median	SD^b^	CV/%^b^	Skew^b^	Kurt^b^
Red soils	Alfisols, ultisols, inceptisols	372	32.98	0.87	33.85	6.97	6.15	3.93	15.5	2.29	9.48
Yellow soils	Alfisols, inceptisols	126	91.26	1.93	93.19	16.56	13.39	14.61	213.3	3.25	13.16
Purple soils	Inceptisols, entisols	84	12.10	0.29	12.38	5.19	4.84	2.36	5.6	0.70	0.28
Limestone soils	Mollisols, inceptisols	22	20.52	1.95	22.47	9.14	9.68	5.04	25.4	0.51	0.92
Skel soils	Inceptisols, entisols	114	36.71	0.10	36.81	4.89	3.30	5.27	27.8	3.56	16.24
Red clay soils	Inceptisols, alfisols	4	2.22	4.80	7.02	5.48	5.04	1.04	1.1	1.92	3.75
Mountain meadow soils	Histosols, inceptisols	4	241.54	37.98	279.52	104.32	49.89	116.98	13680.0	1.98	3.94
Fluvio-aquic soils	Inceptisols, entisols	189	20.34	0.94	21.28	6.38	5.78	3.48	12.1	0.96	1.66
Coastal saline soils	Inceptisols	64	14.25	0.92	15.17	7.37	7.50	3.19	10.2	−0.05	−0.55
Paddy soils	Anthrosols	1175	143.90	1.97	145.87	9.82	8.54	7.13	50.9	8.10	124.50
All profiles		2154	279.42	0.10	279.52	9.06	7.62	9.41	88.6	14.17	346.41

a Reference conversion between Soil Group of the Genetic Soil Classification System of Zhejiang Province and Soil Order of the U.S. Taxonomy.

b N, Min, Max, SD, CV, Skew, Kurt are the abbreviations of the number of soil profiles occurring in a Soil Group, minimum, maximum, standard deviation, coefficient of variation, skewness and kurtosis, respectively.

With soil classification levels up-scaling from Soil Species to Soil Group, the estimated SOC stocks presented small variations with coefficient of variations of 2.1%, 1.4%, 1.4%, and 1.2% for the mean, median, SPS, and PKB methods, respectively. The coefficient of variations of estimated SOC stocks among different methods presented an increasing trend, with 4.4%, 6.2%, 6.3%, and 7.1% for soil classification levels of Soil Species, Soil Family, Subgroup, and Soil Group, respectively. The estimated SOC stocks using the mean, median, and SPS methods at the Soil Species level were 3.1%, 10.6%, and 0.5% lower than that obtained by the PKB method, respectively. The variation of estimated SOC stocks among different methods was obviously larger than that among different soil classification levels.

### Effect of Different Methods on the Estimates of SOC Stocks for Various Soil Groups

Soil Group is the most stable and consistent soil classification level commonly used in China [2]. In this study, the SOC stocks and SOC densities for various Soil Groups estimated by the mean, median, SPS, and PKB methods are presented in Table 3. For the Soil Groups with SOC density values of soil profiles presented positively skewed distribution, the estimated SOC stocks using the median method were usually lower than that using the mean and PKB methods (Table 2, Table 3). The estimated SOC stocks for various Soil Groups using the SPS method were most similar to that obtained by the PKB method because both methods aggregate soil profile properties at the county scale. The estimated SOC stocks for various Soil Groups using the mean and median methods showed large differences compared with the PKB method because both the mean and median methods aggregating soil profile properties at the provincial scale that the SOC density values of all soil profiles belonging to a soil type in the Province were aggregated to one value.

**Table 3:** Estimates of SOC (soil organic carbon) stocks and SOC density values of various Soil Groups using the mean, median, SPS (soil profile statistic), and PKB (pedological professional knowledge based) methods.

Soil Group	Area^a^	PKB		Mean			Median			SPS		
	(km^2^)	SOC density(kg m^−2^)	SOC stock(10^6^ t)	SOC density(kg m^−2^)	SOC stock(10^6^ t)	%^b^	SOC density(kg m^−2^)	SOC stock(10^6^ t)	%^b^	SOC density(kg m^−2^)	SOC stock(10^6^ t)	%^b^
Red soils	39681.3	6.53	259.10	6.73	266.96	3.0	6.24	247.71	−4.4	6.50	258.11	−0.4
Yellow soils	10013.5	16.92	169.45	14.00	140.15	−17.3	12.24	122.54	−27.7	16.72	167.44	−1.2
Purple soils	3597.7	5.73	20.60	5.00	18.01	−12.6	4.77	17.15	−16.7	5.65	20.34	−1.2
Limestone soils	1571.0	10.18	16.00	8.56	13.45	−15.9	9.43	14.82	−7.4	10.47	16.45	2.8
Skel soils	13736.8	5.11	70.20	5.26	72.30	3.0	4.29	58.99	−16.0	5.09	69.95	−0.4
Red clay soils	29.1	5.45	0.16	5.45	0.16	0.0	5.45	0.16	0.0	5.45	0.16	0.0
Mountain meadow soils	3.3	45.30	0.15	104.32	0.34	130.3	49.89	0.16	10.1	45.30	0.15	0.0
Fluvio-aquic soils	4318.3	7.43	32.10	7.33	31.66	−1.4	7.17	30.98	−3.5	7.44	32.13	0.1
Coastal saline soils	2793.3	7.79	21.75	7.58	21.19	−2.6	7.50	20.95	−3.7	7.80	21.78	0.1
Paddy soils	24995.8	9.68	241.98	9.67	241.66	−0.1	9.20	229.86	−5.0	9.64	241.06	−0.4
Total soils	100740.1	8.25	831.49	8.00	805.88	−3.1	7.38	743.32	−10.6	8.21	827.57	−0.5

a Area used for the four methods in this study was from the 1∶50,000 digital soil map.

b Percentage of difference of estimated SOC stocks between the mean, median, or SPS method and the PKB method.

The most pronounced differences of the estimated SOC stocks for the ten Soil Groups (i.e., Red soils, Yellow soils, Purple soils, Limestone soils, Skel soils, Red clay soils, Mountain meadow soils, Fluvio-aquic soils, Coastal saline soils and Paddy soils) among the three GIS-based Soil Type methods occurred on Yellow soils and Mountain meadow soils, which presented high SOC density values with high coefficient of variances. Thus, the process of linking the aggregated SOC density values of soil profiles belonging to these two Soil Groups to corresponding polygons on the 1∶50,000 digital soil map would significantly influence the estimates of SOC stocks. One important factor leading to the significantly high variation of SOC in Mountain meadow soils was the limited number of soil profiles (n = 4) in the soil survey area.

### Effect of Different Methods on the SOC Stock Estimation at Different Soil Depths

**Figure 3:**
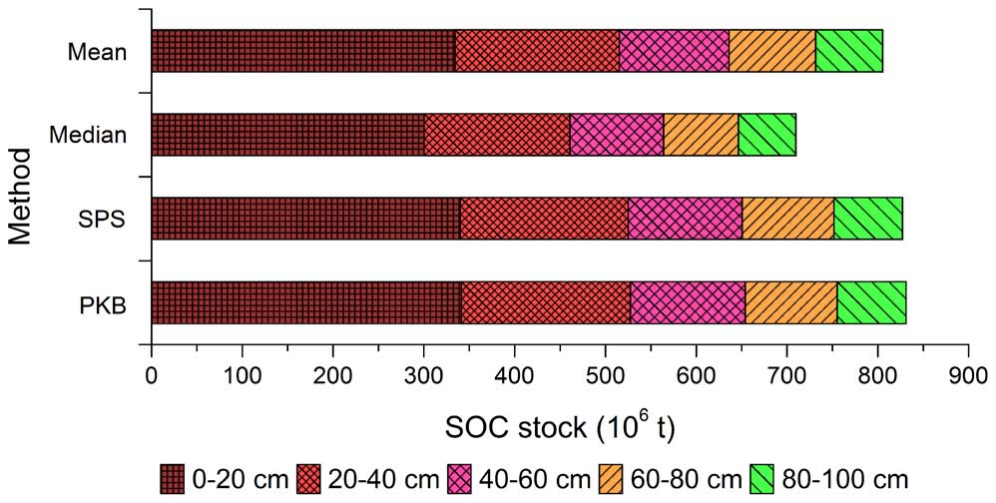
Estimated SOC stocks at different soil depths using the mean, median, SPS (soil profile statistic), and PKB (pedological professional knowledge based) methods.

For the four methods, the trend of estimated SOC stocks at each of the five soil depths (0–20, 20–40, 40–60, 60–80, and 80–100 cm) is consistent with the soil depth of 0–100 cm, followed the order: PKB>SPS>mean>median (Figure 3). At each of the five soil depths, the estimated SOC stock using the SPS method presented the smallest difference while the median method presented the largest difference compared with the PKB method. Decreasing SOC stock with increasing soil depth was evident independent of estimation method, and these decreasing trends are consistent among all four methods. At the depth of 0–20 cm, SOC stocks estimated by the mean, median, SPS, and PKB methods account for 41.5%, 42.3%, 41.1%, and 41.1%, of total SOC stocks, respectively, while the SOC stocks at the depth of 80–100 cm estimated by the four methods account for only 9.2%, 8.9%, 9.1%, and 9.1%, of the total SOC stocks, respectively.

### Comparison of the Differences in the Spatial Patterns of SOC Distribution among the Three GIS-based Soil Type Methods

**Figure 4:**
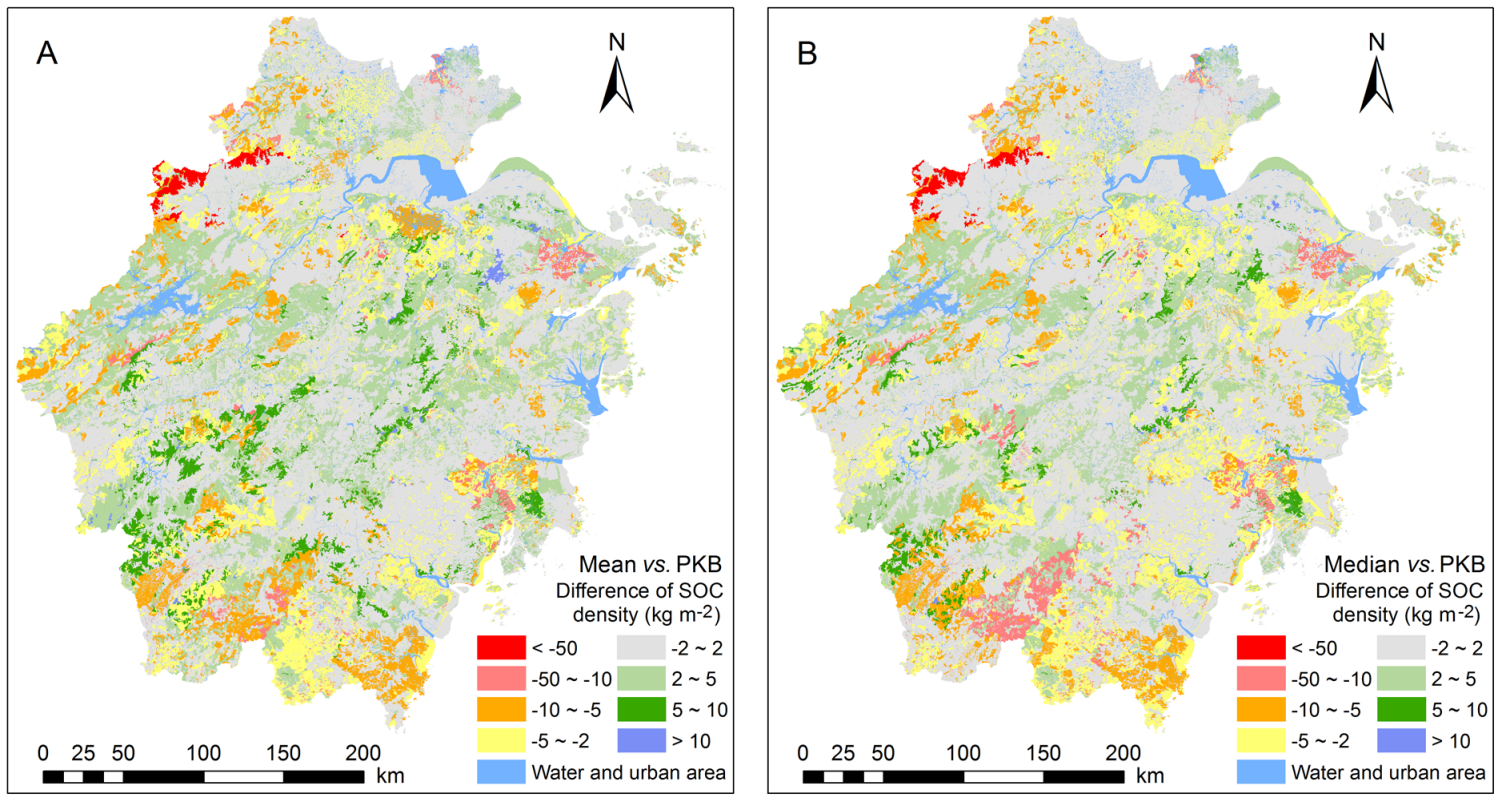
Spatial differences of estimated SOC (soil organic carbon) densities between the mean and PKB (pedological professional knowledge based) methods (A), and between the median and PKB methods (B).

Estimated SOC densities using three GIS-based Soil Type methods showed clearly spatial differences (Figure 4). The largest differences were as high as −229.63 kg m^−2^ between the median and PKB methods and −175.20 kg m^−2^ between the mean and PKB methods. Polygons mostly belonging to the Soil Groups of Mountain meadow soils and Yellow soils showed substantial differences (<−50 kg m^−2^) in SOC densities among the three GIS-based Soil Type methods. For the polygons with large spatial differences in SOC densities ranging from −50 kg m^−2^ to −5 kg m^−2^ and >5 kg m^−2^, the locations were usually in mountain and hilly areas with dominate Soil Groups of Yellow soils, Red soils, and Skew soils, with the remainder in plain areas with dominate Soil Group of Paddy soils. The total areas for spatial differences in SOC densities with absolute value larger than 5 kg m^−2^ between the mean and PKB methods and the median and PKB methods were estimated to be 12883.4 km^2^ and 11428.0 km^2^, accounting for 12.8% and 11.3% of the total area of Zhejiang soils, respectively. The total areas for spatial differences in SOC densities with absolute value lower than 2 kg m^−2^ between the mean and PKB methods and the median and PKB methods were estimated to be 55316.1 km^2^ and 59147.7 km^2^, accounting for 54.9% and 58.7% of the total area of Zhejiang soils, respectively.

## Discussion

With soil classification levels up-scaling from Soil Species to Soil Group, the estimated SOC stock values using the SPS method were most similar to that obtained by the PKB method. The difference of the estimated SOC stocks between the SPS method and the PKB method occurred in the cases where two or more soil profiles belonging to one soil species existed in one county. However, for most cases, there was only one soil profile belonging to one soil species in one county, the SOC density value of that soil profile was used to represent the SOC density value of that soil species in that county, which resulted in the same estimated SOC stock value of that soil species in that county using the SPS and PKB methods. Therefore, more number of soil profiles is needed to further compare the difference between these two methods.

The estimated SOC stock values using the mean method were similar to that using the PKB method. When two or more soil profiles located in the same polygon within a county, the mean SOC density value of these soil profiles was calculated and used to link with that polygon, so the PKB method approaches the mean method in linking SOC density values with polygons to some extent [34], especially for higher soil classification levels (e.g., Soil Group, Subgroup, Soil Family).

With soil profile properties up-scaling from the county scale (the SPS and PKB methods) to the provincial scale (the mean and median methods), the SOC density values of all soil profiles belonging to a soil type in the Province were aggregated to one value. The aggregating procedure for the mean or median method could cause large uncertainties because information about how much area one profile can represent was missing, thus different polygons belonging to the same map unit on the digital soil map were linked with the same SOC density value. For the PKB method, different polygons belonging to the same map unit may be assigned different SOC density values depending on their locations, thus has an obvious advantage in the demonstration of spatial differences in SOC distribution. The difference in aggregating procedure led to Soil Groups (e.g., Mountain meadow soils, Yellow soils) with soil profiles of high variations in SOC density values presented significant spatial differences among the three GIS-based Soil Type methods.

## Conclusions

The up-scaling of soil classification levels from Soil Species to Soil Group has small effect on SOC stock estimation. However, obvious differences occurred with different estimation methods, especially for Soil Groups with high variations in SOC densities and spatial distribution. The PKB method, which links soil profile properties to spatial databases by county, produced more stable results for soil types, soil parent materials, and spatial locations of all soil profiles under consideration. Thus this method has an obvious advantage in the demonstration of differences in spatial patterns of SOC distribution than both the mean and median methods. We recommend the PKB method as a prior option rather than the mean, median, and SPS methods for SOC stock estimation in China, especially when 1∶50,000 soil survey geographic database (soil map) is available. It potentially reduces uncertainties related to up-scaling soil profile properties.
